# Implementation of an Electronic Mental Health Platform for Youth and Young Adults in a School Context Across Alberta, Canada: Thematic Analysis of the Perspectives of Stakeholders

**DOI:** 10.2196/49099

**Published:** 2024-01-17

**Authors:** Gina Dimitropoulos, Emilie M Bassi, Katherine S Bright, Jason Gondziola, Jessica Bradley, Melanie Fersovitch, Leanne Stamp, Haley M LaMonica, Frank Iorfino, Tanya Gaskell, Sara Tomlinson, David Wyatt Johnson

**Affiliations:** 1 Faculty of Social Work University of Calgary Calgary, AB Canada; 2 Calgary Eating Disorders Program Alberta Health Services Calgary, AB Canada; 3 Mathison Centre for Mental Health Research and Education University of Calgary Calgary, AB Canada; 4 Alberta Children's Hospital Research Institute University of Calgary Calgary, AB Canada; 5 School of Nursing and Midwifery Faculty of Health, Community, and Education Mount Royal University Calgary, AB Canada; 6 Heroes in Mind, Advocacy, and Research Consortium (HiMARC) Faculty of Rehabilitation Medicine College of Health Sciences, University of Alberta Edmonton, AB Canada; 7 Provincial Addiction and Mental Health Alberta Health Services Calgary, AB Canada; 8 Brain and Mind Centre University of Sydney Sydney Australia; 9 Departments of Pediatrics Cumming School of Medicine University of Calgary Calgary, AB Canada; 10 Maternal Newborn Child and Youth Strategic Clinical Network Alberta Health Services Calgary, AB Canada

**Keywords:** electronic mental health, eMH, digital mental health, youth and young adult mental health, secondary schools, implementation science, qualitative descriptive methods, mental health platform, mental health, mobile phone

## Abstract

**Background:**

Youth, aged 15 to 24 years, are more likely to experience mental health (MH) or substance use issues than other age groups. This is a critical period for intervention because MH disorders, if left unattended, may become chronic and serious and negatively affect many aspects of a young person’s life. Even among those who are treated, poor outcomes will still occur for a percentage of youth. Electronic MH (eMH) tools have been implemented in traditional MH settings to reach youth requiring assistance with MH and substance use issues. However, the utility of eMH tools in school settings has yet to be investigated.

**Objective:**

The objective of this study was to gain an understanding of the perspectives of key school staff stakeholders regarding barriers and facilitators to the implementation of the Innowell eMH platform in secondary schools across the province of Alberta, Canada.

**Methods:**

Guided by a qualitative descriptive approach, focus groups were conducted to elicit stakeholder perspectives on the perceived implementation challenges and opportunities of embedding the Innowell eMH platform in secondary school MH services. In total, 8 focus groups were conducted with 52 key school staff stakeholders.

**Results:**

Themes related to barriers and facilitators to youth and school MH care professional (MHCP) capacity in implementing and using eMH tools were identified. With respect to youth capacity barriers, the following themes were inductively generated: (1) concerns about some students not being suitable for eMH services, (2) minors requiring consent from parents or caregivers to use eMH services as well as confidentiality and privacy concerns, and (3) limited access to technology and internet service among youth. A second theme related to school MHCP barriers to implementation, which included (1) feeling stretched with high caseloads and change fatigue, (2) concerns with risk and liability, and (3) unmasking MH issues in the face of limited resources. In contrast to the barriers to youth and MHCP capacity, many facilitators to implementation were discussed. Youth capacity facilitators included (1) the potential for youth to be empowered using eMH tools, (2) the platform fostering therapeutic relationships with school personnel, and (3) enhancing access to needed services and resources. MHCP capacity facilitators to implementation were (1) system transformation through flexibility and problem-solving, (2) opportunities for collaboration with youth and MHCPs and across different systems, and (3) an opportunity for the continuity of services.

**Conclusions:**

Our findings highlight nuanced school MHCP perspectives that demonstrate critical youth and MHCP capacity concerns, with consideration for organizational factors that may impede or enhance the implementation processes for embedding eMH in a school context. The barriers and facilitators to implementation provide future researchers and decision makers with challenges and opportunities that could be addressed in the preimplementation phase.

## Introduction

### Background

Globally, mental health (MH) issues are on the rise among young people, particularly since the onset of the COVID-19 pandemic [1,2]. A recent meta-analysis showed that of all people with MH disorders, 62.5% had onset before the age of 25 years [3]. Furthermore, 1 in 10 youths is estimated to experience an MH disorder in their lifetime, including depression, anxiety, and eating disorders [4]. Approximately 1 in 3 youths is estimated to experience an MH disorder in their lifetime [5]. Since the onset of the COVID-19 pandemic, the rates of depression and anxiety are estimated to have doubled among youth compared with the prepandemic period [6]. A recent systematic review found evidence of decreases in access to preventive MH supports coupled with increases in attempted suicide, self-harm, and suicidal ideation among adolescents during the COVID-19 pandemic [2]. The increase is linked to a disruption to daily routines and family connections [4], a reduction in the availability of social support [1], and a lack of necessary coping skills to navigate challenges and stressors experienced by youth [4]. Furthermore, public health restrictions and the increasing rates of social isolation have resulted in higher rates of internet and gaming problems among youth [7,8].

Despite the concerns about the rise in MH problems among youth, many barriers to help-seeking behaviors have been noted, including stigma [9], low MH literacy, and a reluctance to seek MH services [10]. There are many systemic barriers that further interfere with youth accessing needed services when they seek support, including long waitlists and limited availability of evidence-based treatments that are youth friendly and developmentally appropriate [11]. In a recent systematic review, long wait times for MH services were linked to a deterioration in MH outcomes [12]. As a result of the pandemic, web-based care options, including the application of electronic MH (eMH) tools, were widely and rapidly implemented, demonstrating their utility in reaching youth affected by MH and substance misuse and their ability to transcend geographic distance during public health restrictions [13].

The global spike in poor youth MH calls for early detection and intervention of MH disorders using traditional and nontraditional MH services, including eMH services [9,14]. eMH is defined as the “provision of guided mental health care where consumers navigate a rapid and more effective system experience of service entry, skilled assessment, and multidisciplinary and coordinated care, as well as ongoing outcome-based monitoring” [15]. A proliferation of eMH services has been observed using smartphone apps and electronic tools (e-tools) [16,17]. eMH services may supplement various aspects of MH service delivery, including disseminating resources and information, performing ongoing assessments, tracking changes to symptoms and behaviors, delivering psychosocial therapies, and providing peer support [16]. Randomized controlled trials have consistently demonstrated a reduction in some MH symptoms, including mild to moderate depression and anxiety [18-20]. These randomized controlled trials have especially focused on delivering treatment via smartphone interventions (those that provided in-app feedback, and those used to enhance or support face-to-face interventions) [18]. However, research on the implementation of eMH tools outside of routine clinical settings is lacking.

Research suggests the importance of the school setting as a place of first contact for youth who seek MH support [21-23], positioning teachers as the first point of contact to see MH “red flags” [24]. The use of eMH technology provides an opportunity to transform services, including improving the early detection of MH issues, better care coordination and care plans, and referrals to necessary services for youth [25]. However, there is limited research on the implementation and use of eMH tools in school systems.

Existing studies have highlighted general barriers and facilitators to the implementation of digital MH tools among both youth and MH care professionals (MHCPs). Stigma remains a daunting barrier to using eMH services, deterring individuals from seeking help owing to societal and internal biases [26,27]. The quality of the therapeutic relationship, fostered by well-designed interfaces and personalized content, serves as a facilitator, particularly in remote settings [26,27]. Systemic influences (eg, access and infrastructure) can both hinder and facilitate engagement [26]. In addition, one’s level of MH literacy significantly impacts one’s decision to engage, with greater literacy leading to the increased acceptance and use of eMH resources [26]. In summary, these intertwined factors create a complex landscape for eMH and digital MH interventions. The available literature exploring the barriers and facilitators to implementing eMH interventions in the school setting is notably scarce, underscoring the need for a deeper exploration of this important topic. To our knowledge, this study is the first to explore the barriers and facilitators to implementing eMH interventions in the school setting.

**Figure 1:**
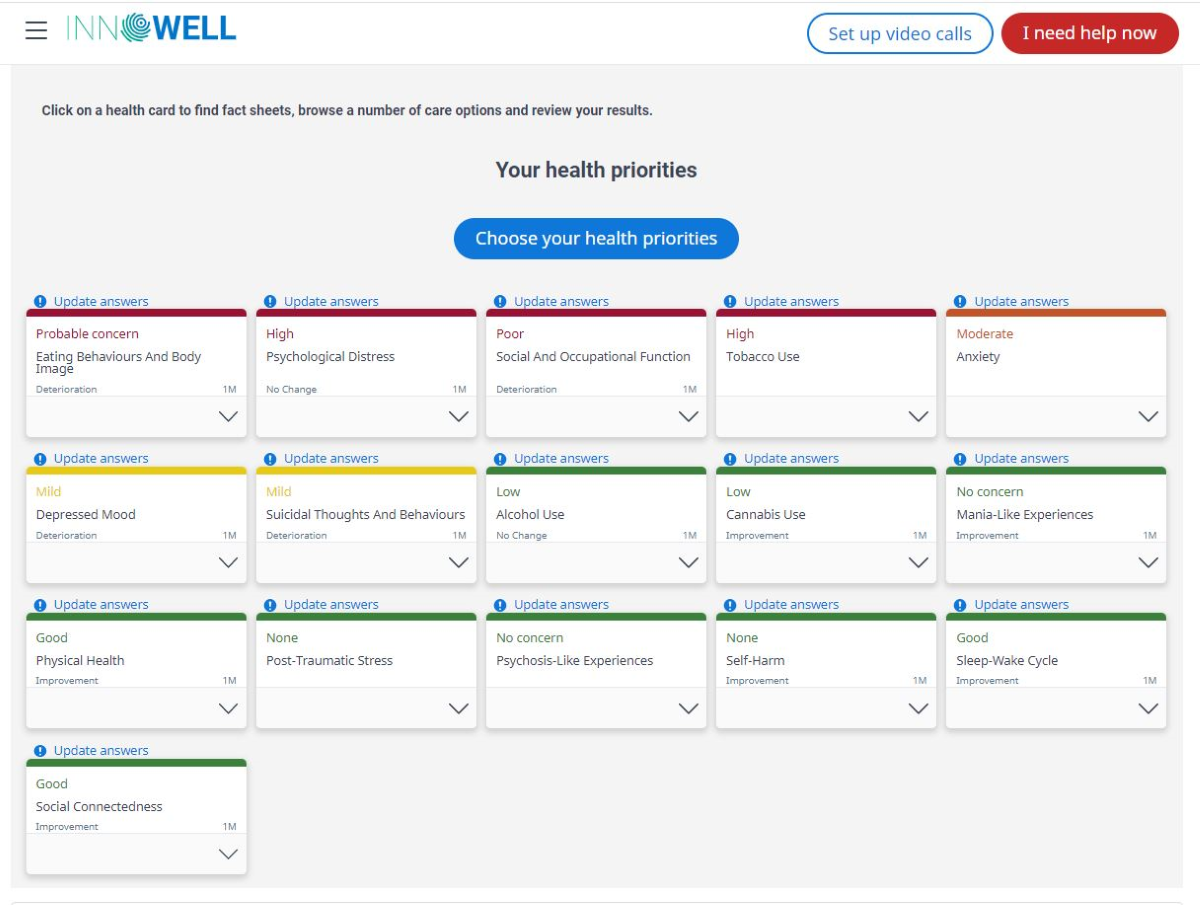
Participant and clinician electronic mental health Innowell platform dashboard health priorities [28].

**Figure 2:**
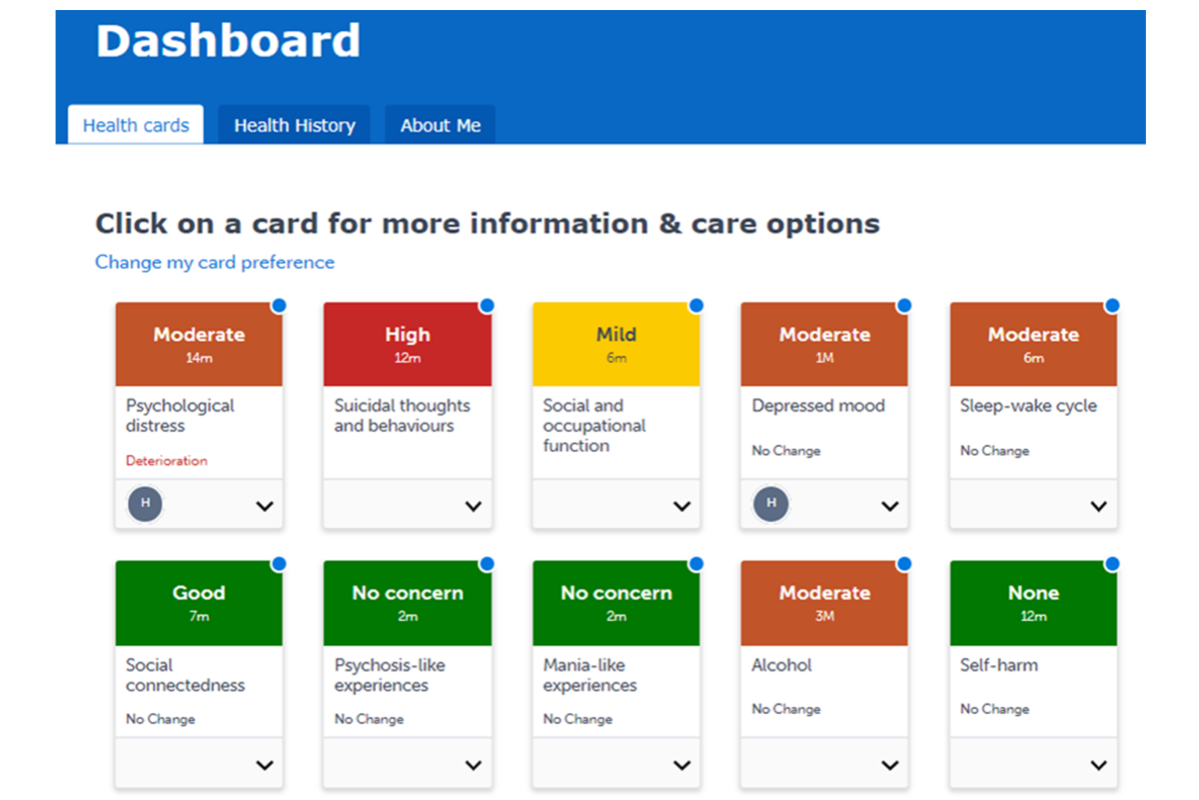
Participant and clinician electronic mental health Innowell platform dashboard health cards [29].

The Innowell eMH platform is a configurable web-based tool that seeks to provide a more personalized approach using measurement-based care to complement existing MH services [25]. The platform empowers youth and young adults to actively participate in their care plan by using measurement-based MH care, including a comprehensive questionnaire consisting of psychometric tools exploring 20 biopsychosocial domains (eg, depression, anxiety, and social connectedness). Examples of health priorities and health cards on the platform’s dashboard are provided in Figure 1 [28] and Figure 2 [29]. After completing baseline measures, platform users are immediately provided access to vetted web-based resources, including apps, e-tools, and crisis line options to use between sessions, and cues to discuss care options with their MHCPs. The platform can assist “with the assessment, feedback, management, and monitoring of their mental ill health and maintenance of well-being by collecting personal and health information from a young person, their clinician(s), and supportive others” [25]. The platform was designed using participatory design in Australia. The participatory design processes included participants with lived experience, health professionals, and service staff from diverse service populations to optimize the usability of the platform design [25,30]. It is important to note that the “platform does not provide stand-alone medical or health advice, risk assessment, clinical diagnosis, or treatment. Instead, it guides and supports (but does not direct) young people and their providers to decide what may be suitable care options” [25].

### Objectives

Hence, it is critical to evaluate the implementation of an eMH tool such as the Innowell eMH platform in secondary schools to assess its acceptability, usability, and efficacy in school contexts. Furthermore, there is a need to examine how eMH tools can be customized for school-based organizations where youth often spend most of their time interacting with educators, guidance counselors, and school MHCPs. This study seeks to elicit the perspectives of key stakeholders within the school divisions regarding their perceptions of barriers and facilitators to the potential implementation of an eMH platform in schools across the province of Alberta, Canada.

## Methods

This study used a qualitative thematic analysis of school stakeholder focus groups (FGs) to better understand their perceived barriers and facilitators to the implementation of an eMH platform for youth and young adults in Alberta.

### Ethical Considerations

This research study received human participant research ethics approval from the University of Calgary Research Ethics Board (project name “Pre-Implementation and Implementation Phase of E-Mental Health for Youth and Young Adults in Alberta”; REB20-1137). Informed consent was completed on the web before the commencement of the FGs. The study participants’ data were deidentified to ensure privacy and confidentiality. Participants were not compensated for their participation.

### Qualitative Descriptive Methodology

A qualitative descriptive methodology was used to describe barriers and facilitators to the implementation of the Innowell eMH platform through the exploration of participants’ perceptions and experiences [31]. This methodology is well suited to health research and is descriptive in nature [31,32], capturing events or conditions from the perspective of individuals and in their everyday language [33]. We sought to obtain a rich description of key stakeholder perspectives rather than researcher-provided interpretations of their preconceived views about the value of an eMH platform. We used an FG approach to obtain data from a group of key stakeholders in schools who may hold diverse concerns about the utility of the platform. In contrast to individual interviews, FGs offer participants the opportunity to share a range of thoughts and to iteratively build on ideas and considerations for integrating an eMH platform in school settings across different geographic regions and diverse student populations [34]. The sample consisted of 52 school district key stakeholders representing 8 communities across Alberta. The web-based FGs were conducted from February 1, 2021 to May 31, 2022.

A semistructured interview guide was created with input from the research and implementation teams on the project. The questions included a focus on thoughts and feelings about the platform and the implementation processes. The interview guide was structured into 3 separate but related sections and was constructed to suit the study objectives, research questions, and the theoretical domains framework (TDF). The TDF is an integrative framework that includes 14 theoretical domains derived from 33 validated health and social psychology theories and 128 constructs that have the potential to drive and explain health-related behavior change [35]. The 14 theoretical domains are knowledge; skills; social or professional role and identity; beliefs about capabilities; optimism; beliefs about consequences; reinforcement; intentions; goals; memory, attention, and decision processes; environmental context and resources; social influences; emotions; and behavioral regulation [36]. The interview guide was designed to explore which TDF domains were relevant for the implementation of the Innowell eMH platform.

FGs were conducted to evaluate the barriers and facilitators that may affect the implementation of the Innowell eMH platform in existing school services. The interview guide covered unique factors about the service setting and organization, unique community contexts that can influence implementation, questions about the unique youth population that the school serves, and the barriers and facilitators that might influence implementation.

The study setting for recruitment included kindergarten to grade 12 schools situated in rural and urban communities across Alberta. This study used a targeted email and web-based meeting recruitment, whereby practice leads from the implementation team working with each community shared recruitment materials with senior decision makers and clinical supervisors at schools taking part in the eMH study. Key stakeholders in this area were recruited, including teachers, administrators, psychologists, school counselors, community connectors, and social workers. The site clinical lead for each community identified potential participants who would be involved in using the platform from a range of different roles, including managers, leaders, and frontline clinicians. Three members of the research team moderated all FGs.

There were 3 inclusion criteria for participants of this study. Each participant had to be employed in the school district as an MHCP, decision maker, educator, or administrator; be proficient in written and spoken English; and use web-based technologies on a laptop computer, desktop computer, tablet, or smartphone.

The FGs were conducted by the research team and included an implementation practice lead, 2 facilitators, and a notetaker who was often a youth research partner. The FGs lasted approximately 90 minutes. Of the 52 participants, 3 (6%) to 10 (19%) participated in each FG. All interviews were audio recorded and transcribed verbatim by a professional transcriber. The transcribed interviews were checked for accuracy by a member of the research team.

A total of 8 FGs were conducted with the 52 key stakeholders from 11 school divisions. The FGs collectively contained representation from 21 schools, including high schools (grades 10-12; n=10, 48%), an elementary school (kindergarten to grade 6; n=1, 5%), combined elementary to high school (kindergarten to grade 12; n=3, 14%), combined junior high to high school (grades 7-12; n=3, 14%), as well as outreach and web-based schools (n=4, 19%). Participants included teachers, administrators, psychologists, school counselors, community connectors, and social workers. Community connectors in the school division are members of the community who support and inform individuals about how to access support groups, services, and information that might help improve their MH and well-being [37]. Sample size was justified at 52 key stakeholders when the research team determined that the sample provided “information power,” a concept used in qualitative methodology to denote that the sample held substantial information [38]. There was representation from public, Catholic, Francophone, and web-based and alternative outreach schools for students (also referred to as clients by some of the participants) who benefit from nontraditional learning methods. The FGs were composed of diverse school district MHCPs, from managers and administrators to teachers.

### Analytic Plan: Thematic Analysis

Using a combined inductive-deductive approach, the research team used the 6 stages of thematic analysis outlined by Braun and Clarke [39]. Thematic analysis is a method of identifying and reporting on themes that emerge through the data [39]. The six-stage analytical process consists of (1) familiarization with the data, (2) coding, (3) generating themes, (4) reviewing themes, (5) defining and naming themes, and (6) producing the report [39].

As part of the project’s youth engagement strategy, a team of coders, including youth and young adults with varying research experience, reviewed the transcribed FGs independently to increase familiarization with the data. After familiarization, the coders conducted a preliminary descriptive coding of their assigned transcripts. The research team generated these codes independently using Microsoft Word and Taguette qualitative coding software. The coders then exchanged FG transcripts and conducted secondary coding. Each transcript was coded by a minimum of 2 coders, which ensured reliability because the codes depended on 2 different individuals achieving and deciding on the same code outcome [40]. The research team then worked collaboratively to group the codes into broader themes through discussion and consensus. Finally, illustrative quotes for each theme were identified. The core research team met regularly to share their impressions of patterns across and within the FG transcripts.

The team closely followed guidelines for publishing qualitative data [41]; for instance, the research team recorded decisions about the coding and the decision-making processes for establishing the themes. The coders worked independently to analyze the data and met regularly to discuss the codes established and assigned. Through consensus, the coding group members formulated their themes based on patterns across the FGs with different school settings. As a form of member checking, our team presented our findings to the broader research team consisting of researchers from different academic institutions to ensure that the data resonated with their experiences and understanding of the academic literature. The practice of reflexivity was used through memoing and group discussions, where members of the research team reflected on their biases and their positionality and questioned their own assumptions throughout the direction of the research process. The practice of keeping written memos was used to record the process and document the rationale for how the data were coded [42].

## Results

### Participant Demographics

Most of the participants (38/52, 73%) identified as women. Many of the participants were counselors or therapists (12/52, 23%) or social workers (11/52, 21%). Of the 52 participants, 19 (37%) were aged between 40 and 49 years, and 32 (62%) had been practicing their profession for ≥11 years. The majority of the participants were born in Canada (45/52, 87%) and held a graduate degree (22/52, 42%). A complete list of descriptive information about the participants is presented in Table 1.

**Table 1:** Descriptive quantitative survey information of school stakeholders (N=52).

Variable			Values, n (%)
**Gender**			
	Woman	38 (73)	
	Man	7 (14)	
	Not reported	7 (13)	
**Profession**			
	Counselor or therapist	12 (23)	
	Social worker	11 (21)	
	Teacher	7 (13)	
	Administrator	6 (12)	
	Psychologist	6 (12)	
	Community Connector	2 (4)	
	Not reported	8 (15)	
**Age group (y)**			
	20-29	3 (6)	
	30-39	15 (29)	
	40-49	19 (37)	
	50-59	8 (15)	
	60-69	2 (4)	
	Not reported	5 (10)	
**Member of a visible minority group**			
	Yes	5 (10)	
	No	39 (75)	
	Not reported	8 (15)	
**Length of time practicing (y)**			
	<1-5	6 (12)	
	6-10	9 (17)	
	≥11	32 (62)	
	Not reported	5 (10)	
**Length of time at organization (y)**			
	<1	6 (12)	
	1-5	10 (19)	
	6-10	9 (17)	
	≥11	22 (42)	
	Not reported	5 (10)	
**Employment status**			
	Full time	35 (67)	
	Part time	7 (13)	
	Other	5 (10)	
	Not reported	5 (10)	
**Ethnicity (n=54)^a^**			
	Indigenous to North America	5 (9)	
	Other North American origins	7 (13)	
	European origins	28 (52)	
	Latin, Central, and South American origins	1 (2)	
	Not reported	11 (21)	
**Born in Canada**			
	Yes	45 (87)	
	No	1 (2)	
	Not reported	6 (12)	
**Level of education**			
	Some university, college, or trades school	1 (2)	
	Completed college or trades school	5 (10)	
	Bachelor’s degree from a university	18 (35)	
	Graduate school	22 (42)	
	Not reported	6 (12)	

^a^Numbers do not equal 52 because participants were allowed to select multiple responses.

### Barriers and Facilitators

In each of the following subsections, we highlight youth and MHCP capacity barriers and facilitators to the implementation of eMH tools in school settings. Youth capacity to engage in eMH services refers to their ability to effectively use digital tools and platforms, such as eMH, for addressing MH concerns. This involves not only their technical skills but also their understanding of the potential benefits and limitations of eMH services. Clinician capacity to engage in eMH services refers to their proficiency in using digital tools and platforms, such as MH assessment tools, to provide MH services to their patients. This also encompasses their ability to navigate the technical aspects of an eMH platform as well as their competence in delivering quality MH care while adhering to the regulations of their profession’s governing body as well as their health care organization’s ethics and regulatory guidelines in the eMH realm.

First, youth capacity barriers were identified, which included the suitability of measurement-based care for all youth with different types of MH disorders that may impact their engagement in eMH services; the role of caregivers, confidentiality, and perceived consent concerns among youth; and inadequate device and internet access. We then established a second theme relating to MHCP capacity barriers to implementation, which included MHCPs feeling stretched with high caseloads and change fatigue, concerns about liability and risk considerations, and the potential to unmask MH issues in the face of service and resource constraints.

By contrast, the final 2 themes consisted of many facilitators to implementation. The third theme—youth capacity facilitators—included the potential for an active and empowered role in care, the potential to foster and enhance therapeutic relationships, and the importance of improving access to services and resources. The fourth theme covered MHCP capacity facilitators to implementation, including the unique flexibility and natural problem-solving skills of school staff that can contribute to system transformation, the potential for collaboration with MHCPs across the continuum and with different systems, and an opportunity to strengthen the continuity of services. Collectively, these themes highlight the potential of the school setting to create opportunities for system transformation through the implementation of eMH tools.

### Barriers to the Implementation of eMH Tools in School Settings

#### Youth Capacity Barriers

##### Individual Characteristics and Implications for the Engagement of Measurement-Based Care

Participants identified numerous factors as potential barriers to youth using the measurement-based care protocol integrated within the Innowell eMH platform. Given the large and diverse youth population served by secondary schools in Alberta, participants expressed concern that some youth with certain types of MH issues and sociodemographic characteristics may be unable to use the Innowell eMH platform. Participants perceived that measurement-based care may not be suitable for youth with the following conditions: severe MH diagnoses (eg, moderate to severe depression or anorexia nervosa), disability diagnoses (eg, attention-deficit/hyperactivity disorder), low cognitive capacities, those experiencing suicidal and self-harm behaviors, and low literacy levels and new or migrating Canadians not fluent in English. Participants were concerned that measurement-based care, which includes the 20-domain assessment features on the Innowell eMH platform, might be lengthy, difficult to understand, and overwhelming:

I do wonder about say students just with their literacy levels, like their comprehension. And especially those who maybe present a specific neurotypical, they present that way but actually maybe their comprehension is really, really weak. And so, onboarding and then going through the diagnostic tools and the self-assessment tools. Like I do wonder about the implications of that.

FG21-P02

This quote demonstrates many participants’ reflections about the implications of onboarding youth who may be unable to easily navigate certain aspects of the platform on their own.

Participants also described a concern that the extensive measurement-based care assessment protocol on the Innowell eMH platform might exacerbate symptoms for some of the youth experiencing complex MH issues:

I do worry that part of this platform, although [it] gives them the tools and the apps and all of those things, and reminds them to speak to the therapist or go see their person. We’re also asking a lot of kids who are struggling...Right? So, they’re not feeling very mentally well and then we’re asking them to do other things to take care of their own wellness. And I worry—are they capable of that? I guess we’ll see.

FG17-P03

This quote demonstrates a perception that some youth may find it difficult to navigate the Innowell eMH platform when they are struggling with MH issues and wellness.

##### Role of Caregivers, Confidentiality, and Caregiver Consent

Other potential barriers identified by participants are problems that may arise in obtaining consent from caregivers and protecting the confidentiality of youth who will access the platform but do not wish to disclose their MH concerns to family members:

And if a lot of the issues surrounds the parent relationship, then that becomes a problem and that has definitely been the past barrier to them getting services sometimes is they don’t want their parents having to sign consents.

FG22-P03

Although not a pervasive issue, participants raised concerns about some youth living in unsafe family environments where it would not be acceptable to disclose an MH issue owing to stigma and fear of reprisals from family members. These factors were described as significant barriers if consent is required from caregivers for the access and use of the Innowell eMH platform. In the absence of clear confidentiality assurances, it may prevent young people from engaging in the apps and e-tools available through the platform and in MH services more broadly.

Information-sharing requirements within the school district with caregivers was also described as a potential barrier to implementation. Participants were concerned about who *owns* the assessment information completed by youth and having to navigate information-sharing requests or requirements from caregivers or other MHCPs:

I think it would need to be very clear what, if any, of the information would be getting shared with their parents, like abundantly clear. I get questions on this all the time when I meet the kids for the first time is, “What exactly is confidentiality?” And many, many questions surrounding that.

FG10-P03

This participant builds on this concern, stating that many young people come to their first session reluctantly, which could spread to minimal uptake of the Innowell eMH platform if information-sharing protocols about consent and parental involvement are unclear. Participants shared that being unclear about confidentiality with young people would be a barrier not only to using the platform but also to young people accessing MH services and resources more generally.

##### Limited Access to Technology and Internet Service Among Youth

A significant barrier described in the school context is a lack of access to technology for youth to use the Innowell eMH platform. With the shift to web-based school and MH services during the COVID-19 pandemic, many MHCPs learned of the access-related challenges with remote and web-based schooling for some youth, especially those residing in remote or rural geographic regions. Participants learned that many youth lack access to technology or devices that would be necessary to use the platform; and many youth also have unstable, or completely lack, Wi-Fi, data, or an internet connection needed to access the platform:

[O]ne of the things that has been a challenge over—specifically with the course of the pandemic, is it really has brought to light how many students—because we are in a rural, northern remote area where Wi-Fi access and device access, cell phone access, all that stuff can be really spotty if not almost impossible for some of our families which means some of the students don’t have access to this kind of thing outside of typical school hours.

FG22-P03

Participants were concerned that introducing an eMH platform, the use of which requires access to devices and a stable Wi-Fi connection, would be an unsuitable initiative for many of their youth population, especially those who cannot afford Wi-Fi access, thus not addressing existing barriers to care for youth considered disadvantaged and vulnerable.

#### MHCP Capacity Barriers

##### Feeling “Really Stretched” With High Caseloads and Change Fatigue

Participants described the school system as often the first point of contact for “a revolving door” (FG17-P02) of students experiencing diverse challenges, from interpersonal problems to the onset of MH disorders. This creates difficulty for them to envision the integration of an eMH platform that might worsen their already high caseloads. Participants described feeling “really stretched” (FG5-P08) and supporting students in the school district as “mentally taxing” (FG21-P02). A participant highlighted these concerns:

[L]ike my vested interest in this point-in-time is it not just a huge, huge source of time and energy suck for them because obviously, before this project, they were busy with their full-time work anyways. Now this is like another thing on their plate. So, that would be my sort of trepidation with this is how it will add more to their plate and potentially increase their stress.

FG21-P02

Participants were concerned about implementing a new eMH platform that would be time consuming and potentially overwhelming to navigate, given the many conflicting demands on their schedules in an ever-changing work environment. Participants also described a reluctance to undergo the necessary organizational changes needed to integrate eMH into their routine practice:

[W]e’re always given new tools constantly. “Try this, these are different assessments and mental health promotions has this going on.”

FG17-P02

Participants questioned how a new tool could be integrated into the existing organizational flow and day-to-day tasks for school staff. This includes the allocation of resources to integrate a new eMH tool, the limited contact time with youth, how this tool factors into waitlist management, and how to integrate this into current data management practices and software. Participants described other data management tools that they have been grappling with in the school system and a concern about how additional eMH tools may create work duplication, especially if there is a lack of interoperability.

Furthermore, the impact of the COVID-19 pandemic on the school system resulted in higher workloads for many school MH staff. Some of the participants noted experiencing a high volume of cases with students with severe MH acuity and a reduction in the availability of community-based MH services; for example, a participant shared how the COVID-19 pandemic has impacted the availability of other community-based MHCPs:

[L]ack of resources. We lost our child psychiatrist in the community. The contract wasn’t renewed. So, that puts an extra burden on the schools for services. That affects both school jurisdictions as well as with the current COVID situation again, it’s the lack of service providers being able to access the school environment.

FG22-P04

Thus, when grappling with external factors, such as the unexpected consequences of the COVID-19 pandemic and the constant upgrading of organizational tools, participants were apprehensive about additional training requirements for another tool to integrate into their daily school routines.

##### MHCP Concerns About Liability

One of the key concerns among participants was the possibility of being liable for the *suicidal thoughts and behaviors* notification embedded in the Innowell eMH platform. Many of the participants shared that they were concerned that a slow response time to a notification that a student is reporting high levels of suicidal thoughts and behaviors may inadvertently prevent timely intervention and therefore contribute to a youth’s suicide. This lag time in responding to a notification of acute crisis could be seen as negligence and thus render the MHCP liable. Participants described a concern that youth might erroneously assume that school professionals are immediately made aware that they are in distress or experiencing an emotional crisis after triggering the suicidal thoughts and behaviors notification:

[B]ut the consistent challenge that happens over and over again is when the school closes at 3:30 PM, our mental health closes at 4:30 PM, what do these parents—so now we know that there’s ideation and we’re at high risk. What do we do in [the community] with these students?

FG22-P02

Thus, participants were concerned about who would be responsible for responding to youth outside of school hours and during school closures, including weekends and summer holidays.

##### Unmasking MH Issues in the Face of Limited Services

Finally, many of the participants expressed concern that they may not have the capacity to respond to the needs of the students within their school district. Some of the participants worried that the increased assessments conducted using measurement-based care embedded within the Innowell eMH platform would unmask the degree to which MH problems impact their student population. Increased assessment would inevitably lead to increased identification of students requiring MH care within student MH services in the school environment. However, participants expressed concern that they would not be able to access needed resources, supports, and specialty services for students presenting with MH concerns owing to long wait times and the absence of evidence-based treatments:

So, to me it’s no different than a lot of rural communities, just a lack of support services to address mental health needs and the negative stigma around mental health and its needs as well.

FG22-P04

Participants were concerned about the potential for this to create a crisis in the school district where MHCPs would be inundated with students with MH problems without proper services and resources to refer them to in the community:

Like I don’t want to say that as “Be careful, let’s not [open the floodgates].” I think we just need to be prepared for that and how are we going to support everybody in the meantime or through that?

FG5-P07

The knowledge of the shortage of primary care services and specialty clinics within the school and across the MH community created significant reservations among participants in considering using the Innowell eMH platform.

### Facilitators to Implementation of eMH Tools in School Settings

#### Youth Capacity Facilitators

##### Creates Potential for an Empowered and Active Role in Care

Participants argued that the most important facilitator to implementation is the belief that the Innowell eMH platform may empower youth to take an active role in their care journey. Being able to access the platform independently, completing assessments that are relevant to them, and accessing apps and e-tools that reflect their needs are key facilitators to implementation. Relatedly, participants acknowledged that young people are far more comfortable using technology, and therefore their readiness to adopt the Innowell eMH platform may be high. Many young people are already using technology to complete and upload homework assignments, schedule appointments, and, in some cases, attend appointments and classes. Thus, participants in our study asserted that there was strong potential for young people to use the platform as an extension of these existing practices.

Participants further suggested that integrating an eMH platform into the school system from primary to secondary schools would allow students to incrementally develop self-management skills and scaffold the information and resources they need for wellness and MH care. Using the Innowell eMH platform within the school context would also allow young people to monitor MH changes over time:

[A] grade 9 student could carry through this platform for the next 4 years and actually manage their own care.

FG5-P04

Collectively, participants viewed the many advantages of eMH technology for their students, including youth empowerment and the ability to acquire self-management skills, resources, and psychoeducation information:

But also, empowering youth. I love the idea that they can access this and it’s at their fingertips and they can start to really see their growth and that’s exciting to me.

FG5-P01

Participants acknowledged youth being able to see their growth over time and access apps and e-tools while in the school system as a salient facilitator to them agreeing to adopt and implement eMH technology in their school. Thus, capitalizing on the young people’s receptivity to technology, embracing their desire to self-manage, and acknowledging this tool as an opportunity to empower youth are all potential implementation enablers.

##### Fosters Therapeutic Relationships

Many of the participants highlighted the important ways in which the Innowell eMH platform can foster a stronger therapeutic relationship between themselves and youth. Participants described several ways in which this can occur, including the potential for youth to develop greater MH literacy, which would lead to awareness and the use of terminology about MH and well-being, leading to a shared language with their MHCP and increased collaboration to explore what care is needed:

[I]t can be a great tool by the looks of it for youth to identify areas. We might know how they’re feeling but not quite able to categorize. “Oh, maybe I’m struggling due to grief,” and being able to recognize, name, and have the language for that, I think, can also be an empowering tool.

FG5-P02

Many of the participants further highlighted that the repeated use of the measures to assess MH issues might be an effective way for youth to alert their MHCP on how they are coping between sessions or how their MH symptoms are changing over time. Participants described the ease of reviewing assessment information that could be updated between sessions and identifying trends that students may not be verbally sharing with their MHCP:

I can see the benefit of it in terms of that—the narrative in between sessions. I often have encouraged the youth that I work with to use the accompanying email to sort of do exactly this piece...there’s a narrative in between things that are happening between sessions, I can’t answer all the time, but you can just send me little notes to say, “This is for next session.” And just sort of in that hopes of creating some sort of form of accountability.

FG8-P01

Participants also anticipated the measurement-based care of the Innowell eMH platform providing information on students’ progress at different time points during the academic year. In a school setting, the platform can facilitate the sharing of information and the strengthening of the therapeutic relationship over many years. The accessibility of the baseline assessments and records of MH changes over time helps inform the students and their MHCP while also increasing the continuity of care.

##### Enhances Access to Services and Resources

The final youth capacity facilitator identified by participants is the opportunity to enhance access to information, resources, wellness apps and e-tools, and web-based support by using the Innowell eMH platform. Closely interconnected to the previous facilitator, participants suggested that youth can learn about, explore, and access resources, services, apps, and e-tools more easily by using the platform:

I feel like this could be beneficial to them because they can kind of access areas that is of interest or is of concern to them and they can get resources right there and then. That they can click on those apps, those different phone numbers or websites are things that I saw when I was clicking on some of those. And I think that that will be great for them.

FG10-P01

Participants recognized that long wait times for specialty services, limited resources, and barriers to accessing MH services, especially in rural areas, might be temporarily addressed by students accessing resources via the Innowell eMH platform.

#### MHCP Capacity Facilitators

##### System Transformation Through Flexibility and Problem-Solving

Participants view the school environment as creating the conditions for them to be early adopters of the Innowell eMH platform because educators are flexible, resilient, and solution focused when faced with challenges:

Yeah. I think our program is unique just in the flexibility that we have...We have the flexibility that, you know, you meet a student, the next day they’re in tears, they can stop by. Or you have a high-risk who could check in in a couple days as opposed to having to wait for 2 weeks.

FG14-P03

Participants suggested that by using the Innowell eMH platform, youth would be monitored more carefully, which is different from what might typically occur at the MH clinics in the community. Many of the participants also brainstormed solutions to the barriers listed in the preceding section; for example, a participant brainstormed solutions for youth who may not have access to devices that allow them to use the Innowell eMH platform and apps:

[E]ven if they don’t have the media devices, there’s nothing wrong with using the Chromebook [in the school] and sitting in the next room and working on it.

FG5-P05

Despite the many barriers delineated by the school personnel regarding the implementation of the Innowell eMH platform, participants frequently brainstormed solutions to some of their unique challenges while participating in the FGs. This speaks to the flexibility and natural problem-solving ability of school personnel to contribute to creating system transformation.

##### Collaboration With MHCPs Across the Continuum and With Different Systems

By developing a comprehensive baseline assessment of students using measurement-based care on the Innowell eMH platform, participants believed that they would develop a better understanding of the care required to meet their students’ unique needs. The information gleaned from the measurement-based care assessment protocol was further perceived as supporting a stepped and staged care approach. This was highlighted by a participant who described how the information could be used:

[F]or like, a clinician’s dashboard—to be able to see those alerts right away would help us really prioritize where we need to target.

FG14-P01

Participants further highlighted that sharing the results of the assessments and ongoing monitoring of MH symptoms with other MHCPs would allow them to share the mutual understanding and language that would enhance collaborations with other MHCPs:

I agree, collaboration and communication is so important. I think being able to get out of our silos and work as a team of professionals will only serve to benefit our young people in need of supports.

FG9-P02

Participants were eager to highlight the potential to remove silos from the MH care system by embracing an eMH platform that could be used across MH care systems. Collectively, many of the MHCPs recognized the potential for these factors to foster a therapeutic relationship with youth, especially if there is increased collaboration among MH teams.

##### Opportunity for the Continuity of Services

Finally, participants shared that the Innowell eMH platform may be used to facilitate the continuity of care across services in the community. Participants highlighted the potential for all members of the care team to share, communicate, and access similar information on the platform about a shared youth client and thus promote the continuity of care. This can help in streamlining the service process by enhancing the continuity of care among services:

‘Cause we want a wraparound service for the kids and I know there’s been lots of privacy issues and that’s kind of been better actually for some clinicians—the more open sharing.

FG9-P04

Some of the participants suggested that MHCPs may embrace the implementation of the Innowell eMH platform as an opportunity to create change within the health care system:

We have a beautiful opportunity here at the middle to drive what happens at the top and at the bottom from a client perspective, our students and our families that’ll be impacted and our community and then our leadership. Our superintendents, our mayors, all of those people. And we right now are the driving middle force that can actually change what’s happening. And we need to utilize this opportunity well.

FG5-P04

Participants of this study strongly advocated that school staff hold a unique role within the community: they establish relationships with a majority of the youth and young adults in every community for many years, while also maintaining relationships with leaders of the school districts and with municipal and provincial levels of government as well, thus providing them with the opportunity to build stronger relationships and drive MH care change by embracing this implementation project.

## Discussion

### Principal Findings

This study aimed to explore perceived barriers and facilitators relating to the implementation of the Innowell eMH platform in secondary schools in Alberta. Using a descriptive qualitative methodology, we held FGs with key stakeholders in school divisions, including administrators, teachers, management staff, school counselors, psychologists, and community connectors (N=52). Our research shows interconnected barriers and facilitators to implementation as it relates to youth and school MHCP capacities, with system-level considerations. We conclude the discussion with a summary of recommendations for addressing implementation in school settings (Textbox 1).

**Recommendations from the qualitative focus groups with school stakeholders.**

**Recommendation and description**

**Clear policies and processes for consent (with regard to accessing mental health [MH] services)**

- School-based leaders and decision makers establish policies and processes regarding consenting mature minors and obtaining and navigating parental consent
- Development or tailoring of existing policies and processes in the local context and culture around the consenting process

**Web-based training environment**

- Create interactive learning activities to enhance educators’ knowledge regarding the application of electronic MH (eMH) tools with diverse students

**Level of education, knowledge, and skills**

- Train school MH care professional (MHCPs) on how to support youth to identify when and how to share MH issues with caregivers, if appropriate
- Establish communities of practice as an approach to provide post training education and supervisory support to ensure that school personnel can apply their knowledge and skills of measurement-based care and eMH tools

**Level of support and supervision**

- Decision makers within school settings identify how to support staff to receive adequate training and supervision to learn to use, and embrace the implementation of, eMH tools and apps
- Ongoing mentorship, supervision, and support is needed to integrate eMH tools into the school settings

**Structure of the school system and contexts of practice**

- Integrated eMH tools fit into established workflows and processes, and work duplication is removed where possible to maximize implementation efforts
- The process of adaption and adoption requires attention to the cultural and contextual components of assessment, formulation, and intervention, including the ways school personnel recognize, explain, and manage distress

**Existing socioeconomic barriers to access**

- Considering socioeconomic status and access in the communities of implementation is a key pillar of equity that should be addressed in the implementation of eMH tools and measurement-based care
- Emphasize the inclusion and integration of local culture beliefs, practices, language, social norms, family, community, and social network for better understanding of help-seeking behaviors

**Address liability concerns and ensure crisis response protocol**

- Liability concerns among stakeholders should be heard, integrated, and rapidly addressed through training and clinical supervision to increase willingness to use eMH tools in school settings
- Ensure that MHCPs have the competencies to effectively respond to a student’s disclosure of suicidal thoughts and behaviors via the Innowell eMH platform
- School administrators and decision makers must establish risk mitigation protocols and procedures to assure school MHCPs that clear pathways are determined and easily implemented to rapidly respond to students experiencing suicidal thoughts and behaviors
- Clinical supervision and administrative support must be made available to school MHCPs requiring assistance with students’ disclosures of suicidal thoughts and behaviors and need of acute care

**Youth focus**

- Keeping youth at the center of eMH implementation strategies could inspire and enliven uptake among MHCPs

**Youth engagement**

- Use eMH tools to enhance and improve the way that youth and MHCPs interact with each other and the way that MH teams from different systems communicate
- Youth should be included in discussions about how to implement eMH in schools

The first theme highlights concern about the extensive assessments embedded within the Innowell eMH platform and youth capacity (eg, attention span and literacy skills) to complete assessment measures. In general, there is concern about whether the literacy level of the Innowell eMH platform matches that of its intended users in the community. In line with our findings, MH status and demographic variables are among the primary user capacity factors described in the literature that can affect eMH use [26,43]. A systematic review by Borghouts et al [43] found 59 studies that reported that the MH status of the user can play a significant role in the engagement and uptake of eMH tools. Although the severity of MH symptoms can be a barrier to some patients using eMH tools [44,45], this may also depend on the type of eMH tool; for example, individuals with more severe MH symptoms may be more likely to use eMH tools inclusive of assessment measures [43,46]. To achieve this, researchers have co-designed a game that trains clinicians to identify how and when to adopt eMH tools with their clients [47]. A similar type of learning activity can be used to enhance educators’ knowledge about the application of eMH tools with diverse students. MHCPs would also benefit from evaluating digital tools through the adoption of adapted rating scales and digital navigators [48].

The results of our study also suggest that school personnel share a concern about how to obtain parental consent and maintain student confidentiality when considering using eMH tools with youth. In keeping with existing research, this finding shows that one of the most significant barriers to young people accessing school MH services is a lack of clarity about confidentiality, especially from their caregivers [10,49]. Young people may avoid accessing school-based MH services and resources if they anticipate negative consequences from their family members [50,51] or if they are required to obtain parental consent [52,53]. This concern also extends to digital MH tools: Cavazos-Rehg et al [51] found that two-thirds of underage youths displaying symptoms of eating disorders were unwilling to obtain parental consent to access a mobile MH intervention. Researchers and clinicians alike strongly advocate that MHCPs use parental consent waivers, reassure young people of their privacy and autonomy, and address adolescent stigma concerns [51] to increase the use of eMH tools. Given the pervasive concerns about confidentiality and the requirement of parental consent to use the Innowell eMH platform in schools, we strongly recommend that school-based leaders and decision makers establish clear policies and processes about consenting mature minors and navigating parental consent. School staff would benefit from informed consent policies, inclusive of teachers, social workers, and administrators that have been approved across school districts in the province or country. We also recommend training school MH personnel on how to support young people to identify when and how to share MH issues with family, if appropriate.

Related to youth capacity barriers, participants were concerned that introducing an eMH platform, the use of which requires access to devices and a stable internet connection, would be unsuitable for many of their youth population, especially for those residing in remote and rural communities. This finding reaffirms the concern about how to integrate eMH technology, given the existing barriers to service access noted in multiple studies [43,54]. Strudwick et al [54] reviewed a total of 31 mobile apps and 114 web-based applications and resources that had the potential to support the MH needs of the broader Canadian population during the COVID-19 pandemic. Key barriers of concern tended to be access, cost, and poor connectivity [54]. Socioeconomic status and access in the communities of implementation are considered key pillars of equity that should be addressed to support the success of future implementation strategies and ensure equitable access of this opportunity [54].

This study points to specific issues and concerns about the lack of available time to build capacity and integrate the Innowell eMH platform into practice. Time constraints, burnout, and change fatigue were also identified as significant barriers to implementation. In alignment with our findings, although high-quality person-centered care is a priority for MH services [55], there are issues pertinent to school MHCPs, such as limited time, competing demands, high caseloads [56], high degrees of burnout [57], and insufficient training and administrative support [58], all of which can create barriers to providing optimal care and adopting a new eMH platform in a system. Furthermore, LaMonica et al [28] argue that if digital solutions are to be successfully used by MH professionals, decision makers must reduce the administrative burden and responsibilities placed on individuals to adopt eMH technology. Importantly, if eMH tools are introduced in an MH service setting, implementation strategies must consider what could be removed or combined to avoid increasing workloads of school MHCPs. We recommend that decision makers within school settings identify how to support staff to receive adequate training and supervision to learn to use, and embrace their curiosity about the implementation of, eMH tools and apps.

From an MHCP capacity perspective, participants expressed concern about how a new eMH platform could be integrated into the existing organizational flow and day-to-day tasks for school MHCPs. If an eMH platform is operationalized into current vision, mission, priorities, and work plans, this is reported to enable implementation and delivery [59]. Operationalization factors that increase implementation are reported to include workflow processes; leadership, including workplace culture and management; and systems, including the organization of people and resources to meet the needs of the community [59]. Greenhalgh et al [60] draw attention to the importance of integrating technological advances in MH care into the work processes and existing tools and resources used by MHCPs. When eMH tools do not fit into traditional workflows and processes, there is a risk of low engagement and poorly sustained implementation once trials end [17,60,61]. Thus, eMH initiatives must fit into the standard workflows of the health system setting [61,62] to improve youth MH outcomes [63]. Work duplication could be removed by reducing administrative burden on professionals by ensuring the interoperability of MH tools with existing management systems (removing the need to enter the same data across multiple systems) [28]. This finding is particularly meaningful for school district professionals who already use existing data management processes, as well as MH tools and resources, and are concerned about the integration of eMH tools into the established organizational workflow. Our findings support the recommendation that eMH tools must fit into established workflows and processes and work duplication removed where possible to maximize implementation efforts.

A major implementation barrier concern among participants is the potential to be held liable for a youth’s suicidal thoughts and behaviors notification alerted through the platform. Although liability concerns have not been sufficiently discussed in the literature, Scott et al [64] examined telehealth policy implications and suggested that some risk should be anticipated and expected in the implementation of eMH tools. Thus, liability concerns among stakeholders should be heard and considered and rapidly addressed through training and clinical supervision to increase willingness to use eMH tools in school settings. Our research sheds light on key liability concerns among school staff that should be considered and urgently addressed during the preimplementation phase to ensure that school MHCPs have the competencies to effectively respond to a student’s disclosure of suicidal thoughts and behaviors via the Innowell eMH platform. This points to a key improvement in quality care by providing young people the opportunity to assess and detect suicidality and, furthermore, empowering the young person to seek support and care pathways for suicidality [65]. Equally important, school administrators and decision makers must establish risk mitigation protocols and procedures to assure school MHCPs that clear pathways are determined and easily implemented to rapidly respond to students experiencing suicidal thoughts and behaviors. A systematic review of MH training for secondary teachers shows that most training interventions have been carried out through facilitated course trainings and workshops, such as MH first aid training, peer support, and suicide prevention and postvention, to name a few [66]. This review showed an improvement in MH knowledge and attitudes among teachers, and the interventions reviewed should be considered in training and preparing schools to implement eMH tools, especially when suicide assessments and alert systems are included in the eMH options. Finally, clinical supervision and administrative support must be made available to school MHCPs requiring assistance with students’ disclosures of suicidal thoughts and behaviors and need of acute care. An area of future research is to review the legal and ethical considerations of telehealth services [64] across different systems to facilitate the successful implementation of eMH tools.

Many of the participants also expressed concern that they may not have the capacity to respond to the needs of the students within their school district owing to limited resources in their school and the broader community. These concerns regarding the availability and accessibility of resources are reported to be challenges with accessing MH services across the globe [67]. Research has shown that school MHCPs tend to have variable or insufficient training coupled with a lack of support from administration, affecting their ability to respond to the diverse needs of students in the school setting [58]. Without the resources available to meet the needs of students who are identified as requiring more MH support, there is great concern that MH symptoms and risk of suicide will worsen [68]. Our findings suggest that early assessment and intervention, including the capacity to respond to the needs of young people and potentially refer them on to additional resources, are critical to the improvement of long-term health and social outcomes [69]. Introducing an eMH platform in a school setting may inevitably lead to the identification of youth who urgently need MH care, validating this concern. Alternatively, the Innowell eMH platform may also identify youth who are able to self-manage through the platform’s apps and electronic resources, preserving current MH services for those individuals in greatest need. Furthermore, gaining an understanding of the number of youth requiring MH services provides a starting point for advocating for increased publicly funded MH services. This advocacy can be done by capitalizing on the surge of interest in digital MH tools through building awareness regarding ways to modernize access to MH services, highlighting evidence of the effectiveness of digital MH tools, and advocating for financial investment [14].

When discussing facilitators to the implementation of eMH tools, participants described the capacity among young people to use technology to access services. We learned that participants view young people as having a strong desire to direct and manage their own care. The ability of young people to take an active role in their own care journey and access resources and information at times that work for them, whether independently or with their MHCP, all highlight the importance of youth capacity as a facilitator to the implementation of the Innowell eMH platform. In line with this finding, Iorfino et al [70] suggest that health systems will see an increased push toward youth owning and managing their own health data to the benefit of both youth and MHCPs. Many studies have addressed the importance of eMH tools as empowering their users by affording more control, choice [71], and shared decision-making [72]. By contrast, MHCPs who are early adopters of digital tools tend to be those who believe that the initiatives will be beneficial to their clients [73]. The scoping review by Hawke et al [74] demonstrated that youth prefer to provide feedback on the care they receive because they have a strong desire to be involved in decision-making. Playing an active role in their care enables young people to cope better, increases their sense of empowerment, and strengthens their connections with health professionals [75]. As MHCPs expressed enthusiasm around the implementation of the Innowell eMH platform, particularly when considering its potential benefits for their students, keeping youth at the center of eMH implementation strategies could inspire and enliven uptake among school MHCPs.

The findings of our study suggest that school MHCPs may use measurement-based care through an eMH platform to identify and monitor how students’ MH concerns are progressing and where new MH problems may be emerging. In fact, our study demonstrated that MHCPs view the measurement-based care protocol embedded in the Innowell eMH platform as providing critical information about a young person’s MH status, a common language for talking about MH concerns, and when changes in MH symptoms are a signal for adaptations in the intensity of services required. This common language as well as the ability to communicate, understand, and interpret their problems and strengths, subjective symptoms, and preferences for care with their MHCP could be established in their collaboration with other MHCPs as well [76]. The changes or lack of changes in the MH symptoms could open collaboration through thoughtful conversations between youth and their MHCP about the direction for care moving forward [77]. To inspire uptake among MHCPs, we support stressing the importance of eMH tools enhancing and improving the way that youth and MHCPs interact with each other and the way that MH teams from different systems communicate, where possible [78].

This study further highlighted organizational implications to the implementation of eMH tools, recognizing the potential for increased access to community-based services and resources and available apps and e-tools. In a scoping review of MH service-level factors for access and engagement for young people, Anderson et al [79] note that choices around resources, increased information, flexible treatment delivery, and person-centered care contributed to young people being engaged in MH services. These benefits also extend to MHCPs and service delivery by helping to better connect young people to the services that they need and providing options to explore when their MHCP is unavailable. Study participants highlighted the potential for all members of the school MH team to share and access similar information about a student to ensure the continuity of care. Furthermore, participants also perceived school MHCPs being involved in the implementation of eMH tools as a turning point for MH services in the community. The unique relationship of school districts with municipal and provincial levels of government was seen as an implementation facilitator that could support significant transformation of youth MH services. Davenport et al [15] demonstrate how primary MH services can be “flipped” using digital health tools. Their work highlights the potential of digital MH services when assessment, triage, and care pathways are created to ensure that young people are matched to the care they need [15]. More recently, the Innowell eMH platform has been used to compare the needs of clients among services and across geographic regions, which can be used to understand the needs of a heterogeneous population and plan services accordingly [80]. This highlights the potential of eMH tools to ensure that youth are accessing the right care at the right time and promoting the continuity of care to avoid the fragmentation of services [81]. Our findings point to the potential of the school setting to support “flipping” youth MH service experiences and outcomes. The school setting is of particular importance, given the unique community-based relationships formed with young people and the potential to participate in deciding the direction of youth MH strategies with local and provincial governments, especially through the implementation of new eMH tools.

### Limitations and Strengths

This study has a few limitations and strengths. Dynamics varied and differed among the FGs, with some groups having more representation from diverse stakeholders than others despite our best efforts to encourage diversity. An open discussion was embraced with each group; therefore, some groups spoke more organically about systemic and organizational implementation challenges than others. A potential limitation is that the clinical lead was often present in the FGs, which may have prevented some people from being forthright about the barriers and enablers specific to their community context as well as their views of the implementation of eMH tools and organizational culture. FGs may also result in certain types of socially acceptable opinions emerging and certain participants dominating. Therefore, this may not be the collective voice we expected. Some strengths of this study that we would like to highlight are the intentional involvement of youth research partners in the analysis of the FG data. This study also recruited from diverse school settings with respect to size, approach, and location (both urban and rural areas in the province). An opportunity for future research would be to target more diverse individuals and ask more targeted questions about organizational and systemic barriers and facilitators experienced by MHCPs and youth, respectively. Future studies could also use multiple methods to increase validity, such as observations and interviews. In addition, this gives way to include young people with lived experiences as participants, which was done as part of this study, and these results will be published in a subsequent manuscript.

### Conclusions

This study sought to explore school MHCPs’ perspectives relating to the implementation of the Innowell eMH platform. Schools are a critical setting to implement eMH tools for youth. Our findings highlight the nuanced perspectives among MHCPs with regard to implementation. Their insights demonstrate critical youth and MHCP concerns, with considerations for organizational-level factors that may impede or enhance the implementation processes for embedding eMH tools in the school context. The identified barriers and facilitators to implementation in a school setting provide future researchers and decision makers with expected (and unexpected) challenges that could be addressed in the preimplementation phase.

## Abbreviations

eMH: electronic mental health
e-tools: electronic tools
FG: focus group
MH: mental health
MHCP: mental health care professional
TDF: theoretical domains framework

## Data Availability

The data generated and analyzed during this study are restricted because the transcripts in entirety could potentially identify participants and are confidential.
